# The Institutional Treatment of Disease

**Published:** 1901-04-20

**Authors:** 


### The Institutional Treatment of Disease.

During recent years the institutional treatment of disease has come to occupy a much more important place in therapeutics than used to be the case. Not only are patients more willing to go to institutions for the sake of undergoing special courses of treatment, but physicians are found to be "ordering" their patients, as the phrase goes, to certain bathing establishments, sanatoria, or other places for this or that kind of treatment, with far greater frequency than they did some years ago, and all this is due to the growing recognition of the fact that, do what one will, it is next to impossible to improvise for each individual patient the apparatus or the attendance which is required for carrying out the various special forms of treatment which can now be obtained with such facility at many institutions. So much has the popularity of certain lines of treatment increased of late years, and so great is the stream of patients which annually sets in towards the better known Continental spas, under the impression that such special forms of treatment as are there in vogue cannot be obtained within the British Isles, that we have thought it would not be without interest if we were to compile for the use of our readers a fairly complete list of the various institutions in these islands where certain largely-used forms of special treatment are available. We therefore offer the following list, and we do so at this particular period of the year because a considerable number of the forms of treatment therein referred to are methods which, although not exclusively appropriate to the summer months are certainly most popular at this time of year.

It must be understood that we do not suggest that anyone could choose a place to go to by inspecting such a list as the following, but we believe that it often happens in practice that a medical man who knows precisely what special treatment is likely to benefit his patient, does not always carry in his mind at the moment the places where such treatment can be obtained, and it is in the hope of serving him as a reminder of the places which are available within this country, and thus of saving him from the too-frequent repetition of the easy-going formula "Go to Nauheim" or "Go to Wildbad"—a prescription which, however appropriate, is sometimes, to say the least of it, unnecessary, seeing how many places are available nearer home; places which are passed over merely because they are out of mind.

### Sanatoria for Consumption.

Among the most important of the institutions devoted to special treatment are the Sanatoria which have lately been established for the cure of consumption by that combination of hygiene, feeding, and general management, which has come to be spoken of as the "Open Air Treatment." Of the advantages of the method there can be no doubt, and although it may be true that each separate detail involved in it could be carried out in a well-appointed home, it is equally true that in practice it is very difficult at home to secure that continuous and prolonged submission to rules of conduct which in an institution follows almost as a matter of course. There are scores of little details which, if accepted as matters of discipline and carried out as part of a general routine, make for recovery; yet for the necessity of which, in regard to any particular individual at any particular time, it might be hard to give a reason; and it is very possible that the daily and hourly carrying out of such apparently trivial but really necessary routine measures—which come so easily as a part of the general discipline of an institution, and yet are so difficult, where, as at home, each act has to be separately ordered and reasoned upon—is responsible for much of the superior efficacy of the sanatorium treatment of consumption.

### Gout and Rheumatism.

Among diseases which are comparatively amenable to the various forms of treatment provided in special institutions, although rebellious to all that is tried for them at home, are the various manifestations of chronic or recurrent gout and "rheumatism," whatever may be meant by that all-embracing term.

In whatever diseases baths, douches, and manipulations are found to be useful forms of treatment, there "institutional" treatment finds its opportunity. It was more especially in the treatment of such conditions that the earlier hydropathic establishments made for themselves their reputation, and with the advances made in recent years it is now more than ever the fact that only at institutions properly fitted up with appliances which it is quite impossible to obtain at home, can hydrotherapy display its full power over disease. Take, for example, the Aix-douche, a most effective treatment, yet one which no house, however elaborate its arrangements, could provide. Nor is it merely a question of appliances. There is no use blinking the fact that by constant practice in one line—narrow as that line may sometimes be—both the medical men by whom the treatment is ordered and the subordinates by whom it is applied, develop a special skill, and are able to obtain results which could hardly be expected from the less precise prescriptions and the less adept applications of less experienced persons.

### Hepatic, Digestive, and Renal Disorders.

Not only are the local derangements, which may be regarded as the results of the rheumatic and gouty processes, successfully treated by hydropathy, but with even more striking effect is an impression sometimes made upon those functional diseases upon which, as we may fairly believe, the more definite manifestations of gout and rheumatism generally depend. By that combination of hydropathy, diet, fresh air, bodily exercise, and mental rest, which is attainable at many a "hydro," results are attainable which are quite out of the reach of the family physician, who, however effectual his medicine, and however good his advice, is quite unable, charm he never so wisely, to get to the root of the mischief, or to alter injurious home habits while the patient remains amid home surroundings.

### The Schott Treatment of Heart Disease.

The general acceptance which the Schott treatment has met with as a most effectual means of giving relief, and sometimes of effecting a cure, in cases of dilatation of the heart, has opened up another field for institutional treatment; for, however perfectly it may be possible to carry out the Nauheim treatment at home in cases in which unlimited means are available, it is only at institutions specially fitted up for the purpose that the majority of patients can obtain the full advantage of the method. In suitable cases the results of a carefully-applied course of Nauheim baths and exercises are so striking that one need not be surprised at the crowds that flock every summer to the little town in the Taunus where this treatment originated. There is, however, but little reason for believing that there is anything in the Nauheim baths, as certainly there is nothing in the Nauheim exercises, that cannot be obtained nearer home, and lately, since the advantages of the treatment have been more generally recognised, many of our English bathing places have specially laid themselves out to provide this form of "cure" for their clients.

### Miscellaneous "Cures."

There are many other conditions in which institutional treatment proves useful beyond those briefly sketched above. For example, there is the "Weir-Mitchell Treatment" in its complete form, as used for cases of anorexia nervosa and nervous exhaustion, and in its modified forms, as so largely employed in America under the name of "rest cure," in cases of neurasthenia and the various results of debility and overwork. This is a treatment which in America is very largely and very effectively used in cases in which it would be almost fair to say that the patients are not really ill at all. But they are run down and overworked, and are on the verge of mental breakdown, and we can hardly doubt that a course of "rest cure" would do far more good to many an overworked head of a house than the traditional "month at the seaside with the children" which is the ordinary form which holiday (!) takes for the lady of the house.

In the treatment of certain constitutional diseases, more especially in prolonged cases of secondary syphilis, treatment by inunctions and by medicated vapour baths is of very great service. Such treatment, however, is very little used in England, chiefly because of the difficulty of carrying it out at home. At institutions provided with the proper appliances, however, all difficulties vanish, and it ought to be just as easy to get these things done in England at such places as Harrogate, and the various hydropathic institutions, as, say, at Aix-la-Chapelle or at Uriage.

Then there are mud baths, and sulphur baths, and hot-air baths, and radiant heat baths, and electric baths, light treatment and X-ray treatment. The two most startling intrusions into our materia medica which have taken place of late years are the scientific application of heat and of light in the treatment of disease. The "light treatment" of lupus and other diseases has so far been mostly used in hospitals and in the private institutions belonging to specialists, but no one who has observed the results which have been obtained can doubt for a moment that there is a great future before it. There are many institutions which ought to be able, while offering that pure air, that regulated exercise and diet, and that orderly life which is found to be most useful in combating the general tendency to tuberculosis, to provide at the same time that local treatment by light which has been found so effectual a means of putting a stop to the progress of lupus; and we can hardly doubt that before long the "light treatment" will become a not inconsiderable branch of institutional treatment in England, where unfortunately lupus is far too common. The application of heat, more especially in cases of rheumatic and other diseases characterised by stiffness, deformity, and pain, has taken many forms; but since it has been shown, more particularly by Mr. Tallerman, that by taking care to maintain dryness of the air surrounding the affected parts much higher temperatures can be employed than had hitherto been considered prudent, new and very striking effects have been obtained. Taking first the treatment by the apparatus invented by Mr. Tallerman, the reports from many hospitals and a large number of private patients leave no doubt as to its surprising efficacy, for details as to which we may refer to Dr. Shadwell's recent work on the subject and to a report by Dr. Neumann on the treatment of sciatica, arthritis deformans, and scleroderma by this system at the Landesbad at Baden-Baden, published in the *Lancet* on March 30th.

Another method of applying heat is that of Mr. Dowsing, in which radiant heat is employed. Many claims have been made at Continental bathing places to the effect that in radiant heat there is some influence beyond and in addition to the effect of mere heat, as conveyed by hot air; but putting these claims on one side there can be no doubt that the Dowsing method of applying radiant heat is very effectual. There are other methods in use, and probably in all alike the secret is to apply a high degree of heat, and at the same time to ensure such a supply of hot dry air as to quickly remove the moisture due to perspiration. But whatever apparatus is employed it is evident enough that skill in its use is an important factor, and thus it is that the hot-air bath is often better applied in an institution than at home. These methods, however, by no means exhaust the list of the many "cures" as they are now called which can be carried out better and more completely in special institutions than at the patient's home. But enough has been said to show that the subject of the institutional treatment of disease is one which cannot be ignored by those who desire to keep abreast of the modern drift of medical practice.

